# Sleeping Sickness

**Published:** 1919-04

**Authors:** 


### Sleeping Sickness.

---

Sleeping sickness has temporarily at least taken the place of influenza as a matter of study for physicians. It is puzzling the profession everywhere and so far the doctors have not been able to find the cause for it or a satisfactory treatment. It does not appear to be limited to any one section or climate and is reported simultaneously from Illinois, Louisiana and many other states. It is claimed by some that it is attributable to influenza, and while most of the cases that have been reported have followed influenza it is by no means certain that influenza is responsible for it. Some of the cases have given way to treatment while others have stubbornly resisted all efforts at cures. The patients are affected in different ways, but it is usually preceded by extreme dizziness and double vision, some patients becoming blind and dumb. A number of cases have been reported in Louisiana, Texas, and Oklahoma, but Illinois and other northern states have so far had the largest number of cases. Dr. Simon Flexner thinks that it has no connection with influenza and that it is not the lethargic encephalitic, or sleeping sickness, of Europe. Will any physicians who have had cases in their practice please report them at once to The Medical Journal, giving the symptoms, treatment and the results for the benefit of others who may have such cases? Even brief reports will do good and may prove of great value to others.

